# High Fat Diets Induce Colonic Epithelial Cell Stress and Inflammation that is Reversed by IL-22

**DOI:** 10.1038/srep28990

**Published:** 2016-06-28

**Authors:** Max Gulhane, Lydia Murray, Rohan Lourie, Hui Tong, Yong H. Sheng, Ran Wang, Alicia Kang, Veronika Schreiber, Kuan Yau Wong, Graham Magor, Stuart Denman, Jakob Begun, Timothy H. Florin, Andrew Perkins, Páraic Ó. Cuív, Michael A. McGuckin, Sumaira Z. Hasnain

**Affiliations:** 1 Immunity, Infection and Inflammation Program, Mater Research Institute - The University of Queensland, Translational Research Institute, Brisbane, Australia; 2 University of Queensland Diamantina Institute, Translational Research Institute, Brisbane, Australia; 3 Blood and Bone Diseases Program, Mater Research Institute - The University of Queensland, Translational Research Institute, Brisbane, Australia; 4 The Commonwealth Scientific and Industrial Research Organization, St Lucia, Brisbane, Australia

## Abstract

Prolonged high fat diets (HFD) induce low-grade chronic intestinal inflammation in mice, and diets high in saturated fat are a risk factor for the development of human inflammatory bowel diseases. We hypothesized that HFD-induced endoplasmic reticulum (ER)/oxidative stress occur in intestinal secretory goblet cells, triggering inflammatory signaling and reducing synthesis/secretion of proteins that form the protective mucus barrier. In cultured intestinal cells non-esterified long-chain saturated fatty acids directly increased oxidative/ER stress leading to protein misfolding. A prolonged HFD elevated the intestinal inflammatory cytokine signature, alongside compromised mucosal barrier integrity with a decrease in goblet cell differentiation and Muc2, a loss in the tight junction protein, claudin-1 and increased serum endotoxin levels. In *Winnie* mice, that develop spontaneous colitis, HFD-feeding increased ER stress, further compromised the mucosal barrier and increased the severity of colitis. In obese mice IL-22 reduced ER/oxidative stress and improved the integrity of the mucosal barrier, and reversed microbial changes associated with obesity with an increase in *Akkermansia muciniphila*. Consistent with epidemiological studies, our experiments suggest that HFDs are likely to impair intestinal barrier function, particularly in early life, which partially involves direct effects of free-fatty acids on intestinal cells, and this can be reversed by IL-22 therapy.

A dense mucus layer prevents inflammation in the intestine by shielding the underlying epithelium from luminal microbes and the external environment. The major macromolecular component of this barrier is the mucin glycoprotein, Muc2, produced by intestinal goblet cells^1^. Muc2 contains cysteine-rich and highly glycosylated domains and requires extensive post-translational modification within the endoplasmic reticulum (ER) and Golgi. The complexity of the mucin protein and the high secretory output of the goblet cell makes Muc2 prone to misfolding, which activates the unfolded protein response (UPR), a cascade of cellular signal transduction events that attempt to restore ER homeostasis. Failure to resolve the misfolding leads to ER stress, which via the UPR can halt protein synthesis in the cell, activate inflammatory signalling and induce apoptosis^2^.

ER stress has been shown to be a component of pathology in many chronic diseases including inflammatory bowel disease. In mice, mutations in the key UPR genes^3^,^4^ and genes involved in correct protein folding lead to intestinal inflammation^5^,^6^. Missense mutations in the *Muc2* gene in *Winnie* and *Eeyore* mice increases Muc2 misfolding in the ER of goblet cells, leading to spontaneous colitis with a complex innate and T_H_17 immune response akin to ulcerative colitis^7^. We have previously shown that specific cytokines can either exacerbate or suppress ER stress and protein production in secretory cells^8^. IL-10 can act directly on goblet cells in the colon to reduce protein misfolding and ER stress and help promote mucus barrier function^9^. More recently, we have identified IL-22 as a potent suppressor of both oxidative and ER stress that acts on secretory pancreatic β-cells to restore secretory protein production under conditions that would normally cause stress and impair protein biosynthesis^8^.

Worldwide prevalence of obesity has increased owing largely to changes in dietary patterns favouring the consumption of high amounts of sugar and saturated fats. Diets high in fat and/or sugar have been shown to induce low-grade intestinal inflammation in mice^10^,^11^,^12^,^13^,^14^, and are also linked to changes in the composition of the gut microbiota^13^,^15^,^16^,^17^, which can be reversed by using intestinal anti-inflammatory agents such as 5-aminosalicylic acid^13^. In mice, high fat diets (HFD) have been shown to exacerbate chemically-induced dextran sodium sulphate (DSS) colitis by up-regulating pro-inflammatory cytokines^18^,^19^,^20^ and exacerbate mucosal tissue damage in mouse models of spontaneous colitis (*Muc2*^−/−^, TNF^ΔARE^ mice)^21^ and ileitis (Mdr1a^−/−^ mice)^22^,^23^. Consistent with this pro-inflammatory response to the HFD, epidemiological studies have implicated high dietary fat intake with an increased risk of IBD^24^. While HFD in mice and obesity in humans appears to diminish intestinal barrier function as measured by passage of endotoxin into the circulation^25^, the effects of a HFD on oxidative/ER stress of goblet cells and intestinal mucin production have not been addressed.

In the present study, we show that HFD-feeding leads to spontaneous goblet cell dysfunction, impaired mucosal barrier function and inflammation, and exacerbates the development of pathology in mice predisposed to ER stress-induced colitis. Mechanistically, we provide the first evidence for a direct role of non-esterified long chain saturated fatty acids in initiating oxidative and ER stress in goblet cells thus impairing the mucus barrier. We also show that HFD-induced intestinal epithelial stress, inflammation and the associated shifts in the microbiome are reversed by IL-22.

## Results

### Long term high fat feeding increases intestinal inflammation, oxidative stress and ER stress

**Figure 1:**
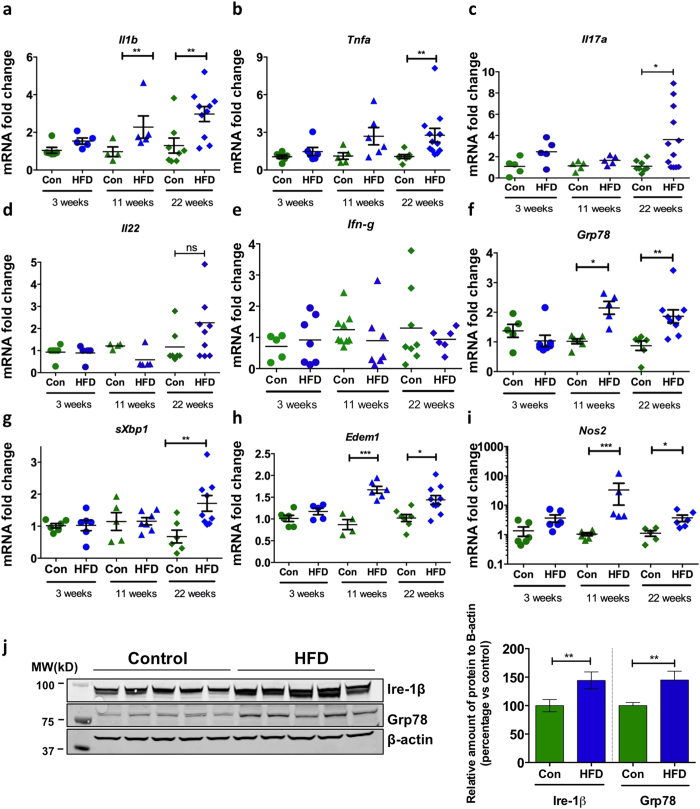
Wild-type C57BL/6 mice were fed a high fat diet (HFD) or normal chow diet (Con) for 3 weeks (n = 6–7 per group), 11 weeks (n = 5–6 per group) or 22 weeks (n = 8–12 per group). Colonic mRNA level of cytokines (**a**) *Il1b*, (**b**) *Tnfα*, (**c**) *Il17a*, (**d**) *Il22* and, (**e**) *Ifn-g,* ER stress markers (**f**) *Grp78*, (**g**) *spliced-Xbp1* and (**h**) *Edem1*, and oxidative stress marker (**i**) *Nos2*, was determined by qRT-PCR in the colon. Normalised to mean expression of *β-*actin and expressed as a fold change compared to in respective control mice. (**j**) SDS-PAGE analysis of epithelial cells isolated from the distal colon of control and HFD mice, immunoblotted with ER stress marker antibodies for Ire-1β and Grp78; β-actin is shown as a loading control. Densitometry shows the quantification as relative amounts of protein of interest normalized against β-actin and presented as a percentage of control samples. n = 5. Mean ± SEM. Unpaired student t test Con versus HFD for each respective experiment duration. *p < 0.05 **p < 0.01 ***p < 0.001.

HFDs have been shown to increase expression of pro-inflammatory cytokines in the colon^10^,^11^,^12^ and some cytokines can directly influence goblet cell mucin biosynthesis^9^ and goblet cell differentiation^26^. To comprehensively analyse the effect of a diet high in saturated fat on the intestine, C57BL/6 mice were fed a HFD (containing 46% of available energy as saturated fat, 20% protein and 4.80% crude fibre) or normal chow diet (containing less than 10% saturated fat, 20% protein and 4.80% crude fibre) for 3, 11 or 22 weeks (Supplementary Fig. 1a). *Il1b* mRNA levels were elevated after 11 weeks of the HFD, whilst levels of *Tnfa* and *Il17a* were only increased after 22 weeks (Fig. 1a–c). However, concentrations of TNFα, IL-1β and IL-17a proteins secreted by cultured mesenteric lymph node leukocytes did not differ between normal chow-fed mice and mice fed a HFD for 22 weeks, in cultured mesenteric lymph node leukocytes (Supplementary Fig. 1b). No changes were observed in the mRNA levels of *Il23*, *Il22*, *Ifng* or in the levels of T_H_2-type cytokines genes *Il13* and *Il4*, which have been reported to induce goblet cell hyperplasia^27^ (Fig. 1d,e; Supplementary Fig. 1). Concomitantly with the mild progressive increased intestinal inflammation, the HFD induced the expression of genes that are markers of ER stress (UPR signalling molecule *sXbp1*, ER chaperone *Grp78* and ERAD chaperone *Edem1*) and oxidative stress (*Nos2* encoding induced nitric oxide synthase) (Fig. 1f–i). Corroborating the increase in gene expression, we also found an increase in ER resident proteins Grp78 and Ire-1β (ER resident endoribonuclease that drives the UPR) proteins in epithelial cells isolated from the distal colon of HFD mice compared to control mice (Fig. 1j).

### High fat diet-induced obesity disrupts mucosal barrier integrity

**Figure 2:**
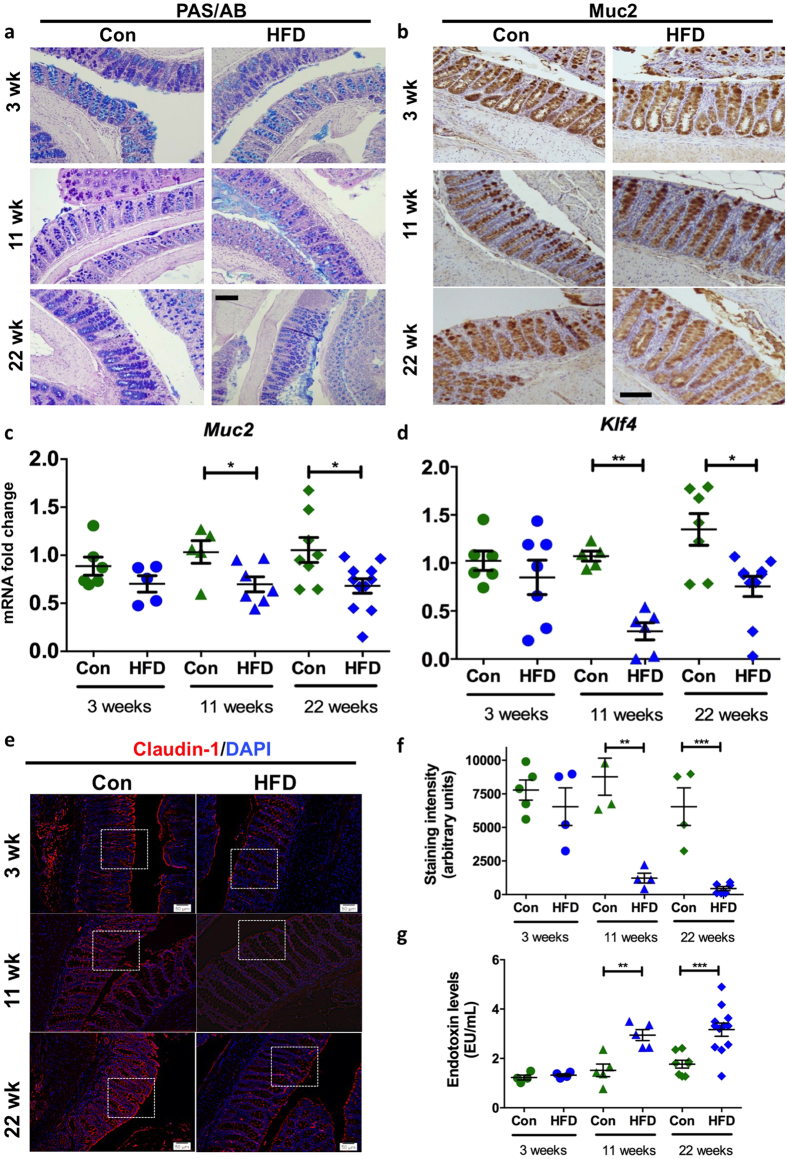
Wild-type C57BL/6 mice were fed a high fat diet (HFD) or normal chow diet (Con) for 3 weeks (n = 6–7 per group), 11 weeks (n = 5–6 per group) or 22 weeks (n = 8–12 per group). (**a**) Periodic Acid Schiff’s-Alcian Blue and (**b**) mature Muc2 immunohistochemical staining shows glycoproteins within the colon in HFD versus Con mice. qRT-PCR was used to determine the colonic mRNA levels of (**c**) *Muc2* and (**d**) *Klf4*. Normalised to mean expression of *β-*actin and expressed as a fold change compared to in respective control mice. (**e**) Immu-nofluorescence was used to determine the levels of claudin-1 (boxes highlight high-powered images shown in Supplementary Fig. 2j), (**f**) shows the staining intensity. (**g**) Serum endotoxin levels (EU/mL). Mean ± SEM. Unpaired student t test Con versus HFD for each respective experiment duration. *p < 0.05 **p < 0.01. Scale bars = 50 μM.

Intestinal goblet cells produce large amounts of complex secretory proteins and therefore are predisposed to high rates of protein misfolding in the ER. Given the induction of markers of ER stress, histological and gene expression analysis was used to ascertain whether HFD-induced inflammation and stress altered the mucosal barrier. No major changes were observed with PAS/Alcian blue staining of all intracellular mucin glycoproteins within goblet cells or immunohistochemical detection of Muc2, which is their major product (Fig. 2a,b; volumetric analyses shown in Supplementary Fig. 2). However, concomitantly with the increased pro-inflammatory cytokines and stress there was a significant reduction in *Muc2* mRNA, which could be explained by the UPR-driven suppression of *Muc2* transcription we have previously described^9^,^28^. Alternatively, this could be explained by reduced goblet cell differentiation. Similar to *Muc2* we observed a decrease in the mRNA of another secreted goblet cell product *Tff3* (Supplementary Fig. 2g), which is key to epithelial restitution after damage and injury^29^.

Therefore, we aimed to explore whether alterations in goblet cell differentiation or goblet cell ER stress could underlie these changes. *Klf4* is a zinc-finger transcription factor required for the terminal differentiation of goblet cells^30^. We observed a significant decrease in *Klf4* expression after 11 weeks and 22 weeks of a HFD (Fig. 2d) and *Spdef*, another transcription factor involved in goblet cell differentiation^9^, was reduced after 22 weeks (Supplementary Fig. 2h). The 22 week HFD led to an increase in accumulation of the non-O-glycosylated Muc2 precursor (Supplementary Fig. 2i), consistent with misfolding of Muc2 in the ER and the increased intestinal expression of ER stress markers. The levels of the integral tight junction protein, claudin-1, also decreased significantly, being 86% and 93% lower after 11 and 22 weeks of the HFD, respectively, with a particular decrease in the apical surface expression (Supplementary Fig. 2j). Overall this is indicative of a disrupted epithelial paracellular barrier (Fig. 2e,f). Moreover, the endotoxin levels in the serum increased following a HFD of 11 and 22 weeks (Fig. 2g), which is consistent with an increase in the permeability of the mucosal barrier.

### HFD exacerbates colitis in the Winnie model of spontaneous ER stress-induced colitis

**Figure 3:**
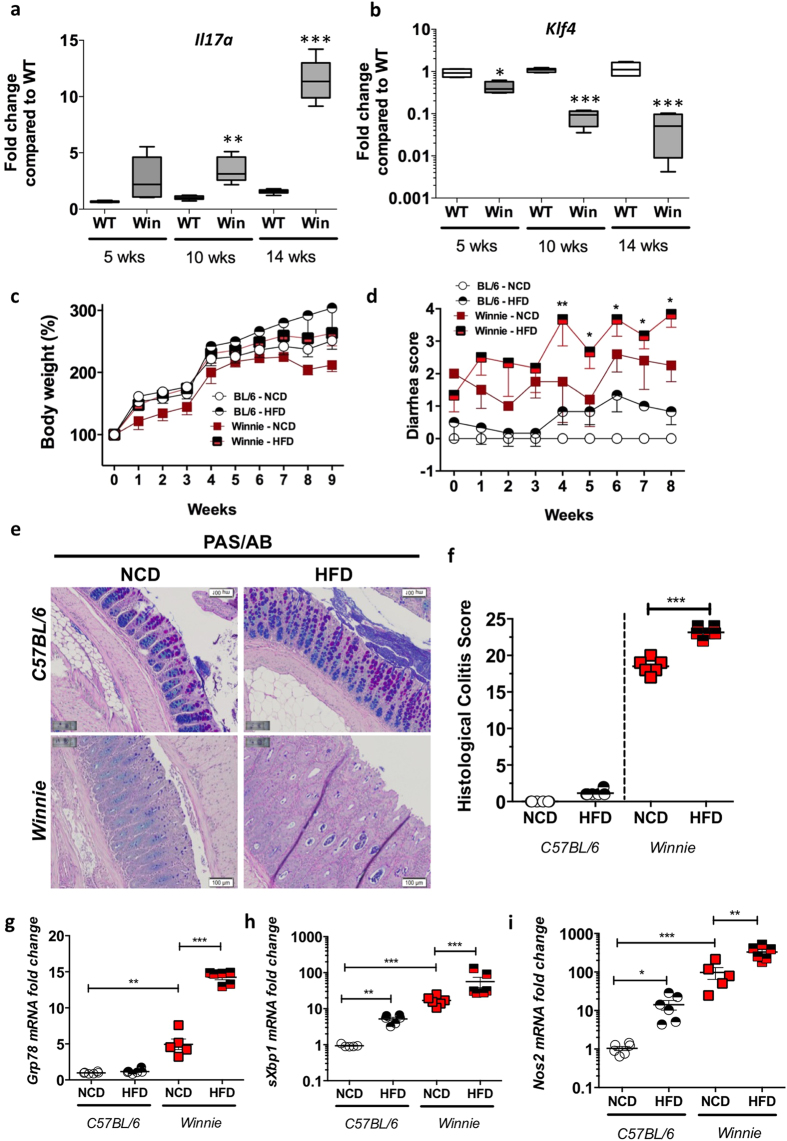
qRT-PCR was used to determine the levels of (**a**) *Il17a* and (**b**) *Klf4* in wild-type C57BL/6 (Con) or *Winnie* (Win) mice with age. (**c**) Con or *Winnie* mice were fed a high fat diet (HFD) or regular control diet (NCD) for 9 weeks following weaning (3 weeks of age); body weight changes across the duration of experiment. (**d**) Diarrhea score during the course of the experiment. (**e**) Periodic Acid-Schiff’s-Alcian Blue staining and (**f**) histological score in wild-type and *Winnie* mice on NCD or HFD. qRT-PCR was used to determine the levels of ER stress markers (**g**) *Grp78* and (**h**) *sXbp1* and oxidative stress marker (**i**) *Nos2*. n = 5–8 per group. qRT-PCR data is normalised to mean expression of *β-*actin and expressed as a fold change compared to in respective control mice. Mean ± SEM or box plots with whiskers show median, Q1, Q3 and min/max One way ANOVA with Bonferroni post-test Con versus HFD, Con versus Win, Con versus Win HFD and Win versus Win HFD. *p < 0.05 **p < 0.01 ***p < 0.001. Scale bars = 100 μM.

In *Winnie* mice the missense mutation in the gene encoding *Muc2* results in the misfolding of Muc2 precursor that accumulates within the ER, resulting in the initiation of the UPR, and decreased Muc2 biosynthesis leading to a diminished mucus barrier and intestinal inflammation^7^. Therefore, the *Winnie* colitis results from an epithelial defect with a normal underlying immune system, rather than the major disturbances to immunity seen in many other mouse models of inflammatory bowel disease. *Winnie* mice progressively develop more severe colitis with age, which is accompanied by inflammation exemplified by progressively increasing *Il17a* (Fig. 3a). We have previously reported decreased *Muc2* mRNA expression^31^ and now report a decrease in *Klf4* (Fig. 3b). We hypothesised that a HFD would exacerbate ER stress and the severity of developing colitis and therefore fed *Winnie* mice from weaning with either a HFD or normal chow diet (NCD). On the NCD *Winnie* mice gained slightly less weight than WT mice, but both WT and *Winnie* mice gained more weight when placed on the HFD from weaning (Fig. 3c). Interestingly, after 4 weeks of the HFD *Winnie* mice had significantly increased diarrhea scores compared to their *Winnie* littermates on a NCD (Fig. 3d), and also had consistently bloody faeces, therefore the study was terminated after 9 weeks. While there were no differences in the colon weight/length between *Winnie* mice fed NCD and HFD (Supplementary Fig. 3), *Winnie* mice fed a HFD had more severe and widespread colonic damage with prominent mucosal erosions and crypt abscesses (Fig. 3e).

**Figure 4:**
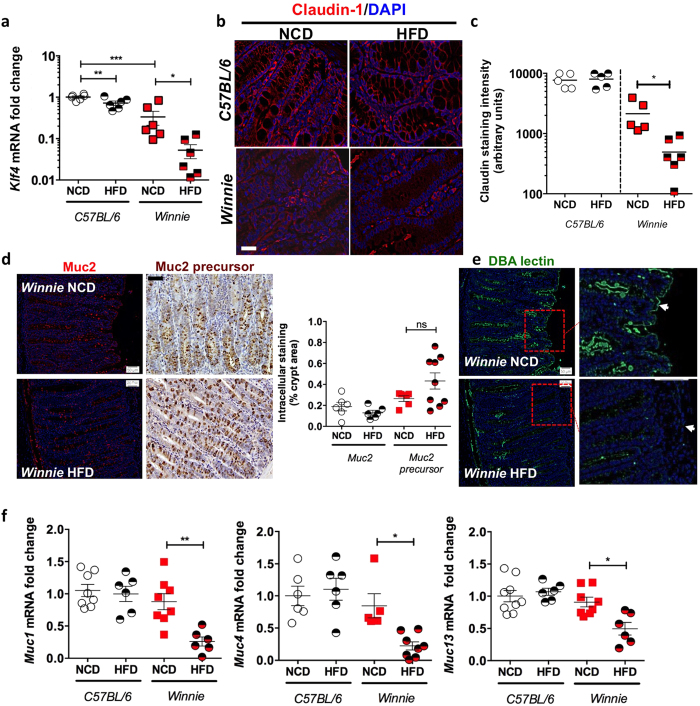
qRT-PCR was used to determine the levels of (**a**) *Klf4* in Con or *Winnie* mice fed a high fat diet (HFD) or regular control diet (NCD) for 9 weeks following weaning (3 weeks of age). (**b**) Immunofluorescence staining with claudin-1 antibody and (**c**) staining intensity as determined by ImageJ analysis. (**d**) Immunofluorescence/immunohistochemical staining was used to determine the levels of mature Muc2 and Muc2 precursor respectively in *Winnie* mice on control and high fat diet; % of intracellular staining per crypt area is shown as box and whisker plots. (**e**) DBA lectin staining was used to determine the changes in the glycocalyx in Winnie mice on a HFD. (**f**) qRT-PCR was used to determine the mRNA expression of genes encoding cell-surface mucins, *Muc1*, *Muc4* and *Muc13*. n = 5–7 per group. Normalised to mean expression of *β-*actin and expressed as a fold change compared to in respective control mice. Mean ± SEM. One way ANOVA with Bonferroni post-test Con versus HFD, Con versus Win, Con versus Win HFD and Win versus Win HFD. *p < 0.05 **p < 0.01 ***p < 0.001. Scale bars = 50 μM.

Mirroring the histological data, a clear increase in ER stress markers (*Grp78* and *sXbp1*), oxidative stress marker (*Nos2*) and a decrease in goblet cell differentiation (*Klf4*) was observed in the *Winnie* mice fed the HFD (Fig. 3g–i, Fig. 4a). Diminished claudin-1 protein staining was observed in the *Winnie* vs WT mice, and this was further reduced by the HFD (Fig. 4b,c). Immunohistochemical staining with antibodies against mature Muc2 and the non-glycosylated Muc2 precursor revealed a trend towards decreased mature Muc2 and increased Muc2 precursor in *Winnie* mice on a HFD, although this result did not reach significance (Fig. 4d). This suggests that in the face of existing inflammation and ER stress a HFD increases ER stress, mucosal barrier disruption and mucosal inflammation.

To further corroborate this notion, we used immunofluorescence with DBA lectin, which detects an O-linked sugar αD-GalNAc. Staining with the DBA lectin revealed that very few goblet cells within the intestinal crypts had positive staining in the *Winnie* mice kept on a HFD (Fig. 4e). Moreover, we noticed diminished O-glycans in the glycocalyx, which is the carbohydrate-rich zone found at the apical epithelial surface, consisting of glycoproteins including cell surface mucins that project into the intestinal lumen (Fig. 4e; highlighted by white arrows). Consistent with decreased glycocalyx glycans, we saw a significant reduction in the mRNA of genes encoding the cell surface mucins, *Muc1, Muc4* and *Muc13* in *Winnie* mice kept on a HFD (Fig. 4f).

### Non-esterified fatty acids but not short chain fatty acids cause stress and protein misfolding in intestinal epithelial cells

**Figure 5:**
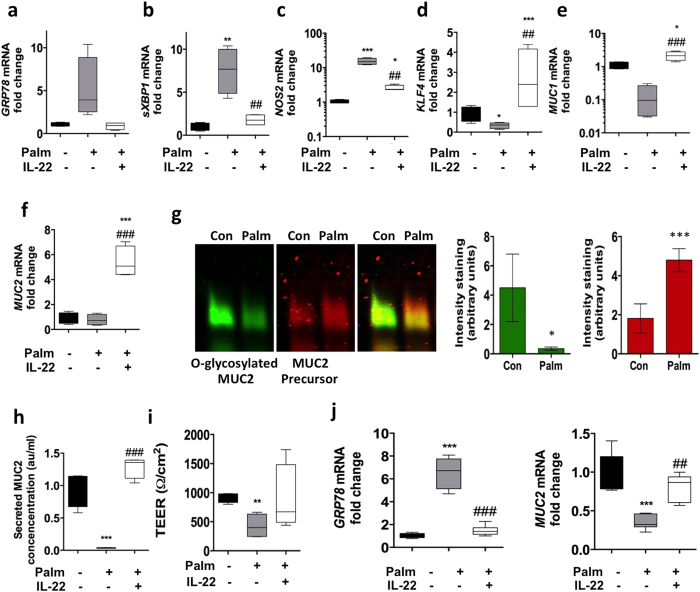
LS174T cells were treated with control BSA, 0.5 mM palmitate or 0.5 mM palmitate and 50 ng/mL of IL-22 for 24 hours. mRNA levels of ER/oxidative stress markers (**a**) *GRP78*, (**b**) *spliced XBP1* (**c**) *NOS2*, (**d**) goblet cell differentiation factor *KLF4*, (**e**) component of the glycocalyx cell surface *MUC1*, (**f**) major secretory product of goblet cells *MUC2* were determined by qRT-PCR. Normalized to expression of *β-Actin* and expressed as a fold change of the mean of BSA controls. Statistics: n = 8 per group (2 individual experiments). Data presented as box plots with whiskers show median, Q1, Q3 and min/max. One-way ANOVA with Bonferroni post-test Con versus treatments. *P < 0.05 **P < 0.01 ***P < 0.001. (**g**) Identification of the MUC2 precursor and mature glycosylated proteins in cell lysates analyzed under reducing conditions by agarose gel electrophoresis and Western blotting with the human MUC2 antibody reactive with glycosylated MUC2 and 4F1 MUC2 nonglycosylated precursor antibody, densitometry shows n = 4–6 from 2 independent experiments, mean ± SEM. Unpaired student t test Con versus treatments; *P < 0.05 ***P < 0.001. Secreted MUC2 concentration shown as arbitrary units/mL were determined using an ELISA. n = 8. (**i**) Transepithelial electrical resistance was measured in control and treated cells after 24 h. qRT-PCR was used to determine the changes in the mRNA levels of ER stress marker (**j**) *GRP78* and secreted gel-forming *MUCIN-2* in human intestinal primary organoid cultures treated with control BSA, 0.5 mM palmitate or 0.5 mM palmitate and 50 ng/mL of IL-22 for 24 hours. n = 6. Data presented as box plots with whiskers show median, Q1, Q3 and min/max. One-way ANOVA with Bonferroni post-test Con versus treatments. *p < 0.05 **p < 0.01 ***p < 0.001.

A prolonged HFD resulted in increased ER stress in the intestine in normal mice, and accentuated intestinal ER stress and inflammation in *Winnie* mice. In obesity and diabetes, non-esterified fatty acids which are a component of the high fat diet, are important contributors to oxidative and ER stress in pancreatic β-cells, which are insulin-producing secretory cells. We observe an increase in circulating serum triglyceride levels with the long term high fat feeding (Supplementary Fig. 4a). Therefore, we wanted to determine whether non-esterified fatty acids could directly initiate protein misfolding and ER stress in intestinal epithelial cells and compare their effects to short chain fatty acids, which, in contrast, have been reported to promote mucosal barrier integrity. To this end, we used an *in-vitro* model of intestinal goblet cells, the human colonic LS174T cells that contain secretory cells predominantly producing MUC2^9^. Palmitate treatment induced significant stress in LS174T cells with an increase in s*XBP1* and *NOS2* (Fig. 5a–c). In contrast, the short chain fatty acids: butyrate, acetate and propionate did not affect ER stress or oxidative stress (Supplementary Fig. 4). The goblet cell differentiation transcription factor *KLF4* and the *MUC1* cell surface mucin were significantly reduced by palmitate treatment. In contrast, however, mRNA levels of the gel-forming mucin *MUC2* remained unaltered with palmitate treatment (Fig. 5e,f), whereas short chain fatty acids significantly upregulated *MUC2* corroborating previous reports (Supplementary Fig. 4e)^32^. Although there were no changes observed in *MUC2* mRNA, because of the induction of ER stress we examined MUC2 protein biosynthesis. Palmitate reduced production of mature fully glycosylated MUC2 accompanied by increased non-glycosylated MUC2 precursor and a reduction in mature MUC2 secretion, providing evidence of protein misfolding consistent with the UPR activation demonstrated by PCR (Fig. 5g,h). In line with these findings, we also saw a significant reduction in the transepithelial electric resistance (quantitative measure of the integrity of tight junctions) after 24 h of palmitate exposure (Fig. 5i). Using primary human intestinal colonic crypt cultures we confirmed the direct effect of palmitate on ER stress and also observed a decrease in *MUC2* mRNA levels not seen in the LS174T cells (Fig. 5j). These results show that palmitate results in oxidative and ER stress resulting in misfolding and aggregation of MUC2, and decreased production of mucin.

### IL-22 suppresses stress caused by non-esterified fatty acids (NEFAs) in intestinal epithelial cells

We have recently demonstrated that IL-22 can potently suppress oxidative and ER stress in pancreatic β-cells by upregulating key genes in the antioxidant pathway and suppressing genes involved in producing reactive oxygen and nitrogen species, thereby restoring high quality insulin secretion and resolving obesity-induced hyperglycaemia^8^. Therefore, we examined whether IL-22 can suppress palmitate-induced stress *in vitro* in the LS174T cells. IL-22 alone did not induce any ER stress as GRP78 and sXBP1 remained unaltered (Supplementary Fig. 5b,c). Importantly, IL-22 even in the absence of stress led to a decrease in pro-oxidant gene, *NOS2,* in LS174T cells (Supplementary Fig. 5g), similar to our observations in pancreatic beta cells^8^. Corroborating the gene expression changes in oxidative stress, the Griess assay demonstrated that IL-22 reduced nitrite (stable metabolite of nitric oxide) concentrations below the baseline in untreated control cells (Supplementary Fig. 5h). Cells treated with palmitate and IL-22 concomitantly had significantly reduced ER stress and oxidative stress (Fig. 5a–c), which led to a recovery of goblet cell differentiation factors, *MUC1* and *MUC2* mRNA expression (Fig. 5d–f) and MUC2 secretion (Fig. 5g,h). Using primary human intestinal colonic crypt cultures we confirmed that IL-22 suppressed ER stress induced by palmitate and resulted in the recovery of the *MUC2* mRNA (Fig. 5j).

### IL-22 decreases obesity-induced intestinal stress and inflammation to restore intestinal barrier function

**Figure 6:**
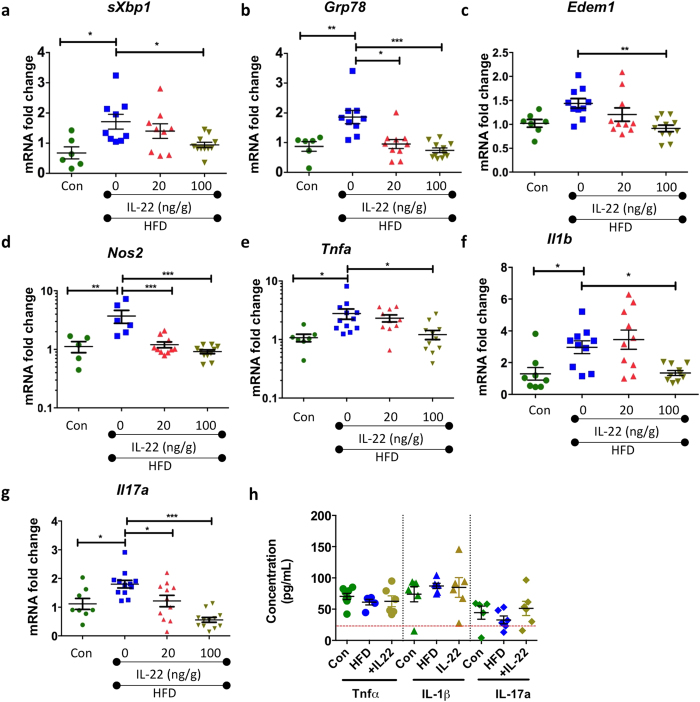
Wild-type C57BL/6 mice were fed a high fat diet (HFD) or normal chow diet (Con) for 22 weeks. After 18 weeks, recombinant IL-22 was administered at 20 ng/g or 100 ng/g i.p twice weekly for 4 weeks. Expression of genes, using qRT-PCR, encoding ER stress markers (**a**) *sXbp1*, (**b**) *Grp78* and (**c**) *Edem1*, (**d**) oxidative stress marker *Nos2*. Gene expression of proinflammatory cytokines (**e**) *Tnfa* (**f**) *Il1b* and, (**g**) *Il17a* was determined by qRT-PCR and ELISA was used to determine cytokine protein levels of TNF-α, IL-1β and IL17-a secreted by anti-CD3/anti-CD45–stimulated leukocytes isolated from mesenteric lymph nodes. qRT-PCR data is normalized to expression of *β-Actin* and expressed as a fold change of the mean of control. Mean ± SEM. N = 8–12 animals per group. One-Way ANOVA with Bonferroni post-test Con versus HFD and HFD versus IL-22 treatment. *p < 0.05 **p < 0.01 ***p < 0.001.

Given the increase in intestinal ER stress and oxidative stress as well as elevated expression of intestinal pro-inflammatory cytokines during obesity (after 22 weeks of HFD), we sought to determine whether administration of recombinant IL-22 could resolve mucosal barrier stress and inflammation. In a previously published experiment mice were fed a HFD for 18 weeks and then treated twice weekly with either 20 or 100 ng/g IL-22 for 4 weeks. We previously reported that both groups treated with IL-22 progressively lost weight, quickly regained glycemic control and 4 weeks of treatment also restored insulin sensitivity^8^. While no major changes were observed in the colon weight or length (ratio shown in Supplementary Fig. 5a) or in the blind scoring for the histological appearance of the intestine (data not shown), IL-22 reduced intestinal ER stress (*sXbp1*, *Grp78* and *Edem1*) in a dose-dependent manner (Fig. 6a–c; Supplementary Fig. 5f shows Grp78 protein). Therefore, we investigated whether decreased ER stress in response to IL-22 treatment was accompanied by decreased oxidative stress and inflammation and found obesity-induced intestinal *Nos2, Il1b, Tnfa* and *Il17a* mRNA expression was markedly reduced by IL-22, with evidence for a dose dependent effect (Fig. 6d–g). Importantly, these alterations in cytokines occurred locally in the intestinal mucosa, as no changes were observed in secretion of TNF-α, IL-1β or IL-17a by cultured mesenteric lymph node leukocytes from mice with and without HFD-feeding or IL-22 treatment (Fig. 6h).

**Figure 7:**
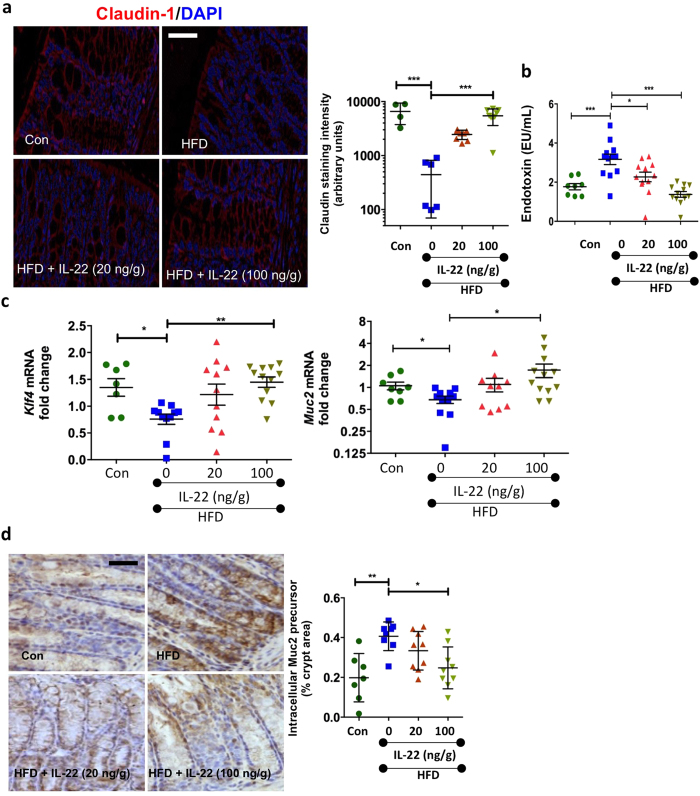
Immunofluorescent staining with (**a**) the claudin-1 antibody in wild-type C57BL/6 mice fed a high fat diet (HFD) or normal chow diet (Con) for 22 weeks and treated with recombinant IL-22 20 ng/g or 100 ng/g i.p twice weekly) for the last 4 weeks; staining intensity as determined by ImageJ analysis. (**b**) Endotoxin levels (EU/mL) in the serum collected from mice at the end of the experiment. (**c**) Levels of goblet cell transcription factor *Klf4* and goblet cell mucin *Muc2* were determined by qRT-PCR. Normalized to expression of *β-Actin* and expressed as a fold change of the mean of control. (**d**) Immunohistochemical staining for Muc2 precursor and quantification as a percentage of total crypt area using ImageJ software. Data presented are mean ± SEM. N = 8–12 animals per group. One-Way ANOVA with Bonferroni post-test Con versus HFD and HFD versus IL-22 treatment. *p < 0.05 **p < 0.01 ***p < 0.001. Scale bars = 50 μM.

Since impaired mucus production and obesity-driven intestinal inflammation are the likely mechanisms increasing permeability of the mucosal barrier and systemic endotoxin levels^33^,^34^ (Fig. 2), we hypothesised that in obese mice treated with IL-22, reduced ER stress and inflammation would lead to improved intestinal barrier function. IL-22 treatment induced a significant increase in claudin-1 staining in a dose-dependent manner (Fig. 7a), which coincided with a decrease in endotoxin levels in the serum (Fig. 7b). We noted a slight increase in crypt length in the mid-colon with the HFD which was reduced with IL-22 (Supplementary Figure 5c). However, our assessment of proliferation by using Ki67 (Supplementary Fig. 5d) and apoptosis using TUNEL staining (Supplementary Fig. 4e) showed no major changes in the turnover of epithelial cells with the HFD or with IL-22 treatment. There was an increase in *Klf4* and *Muc2* mRNA levels, consistent with an improvement in ER function and the resolution of the UPR-driven decrease in *Muc2* transcription (Fig. 7c)^9^. Consistent with earlier experiments (Fig. 2), the HFD did not alter goblet cell size or intracellular Muc2 protein, which also remained unchanged with either dose of IL-22 (Supplementary Fig. 5b). Obesity-induced misfolding of Muc2 (as determined by Muc2 precursor staining) was reduced following treatment with the higher IL-22 dose (Fig. 7d).

### Shifts in HFD-induced changes in intestinal bacterial population are reversed with IL-22 treatment

**Figure 8:**
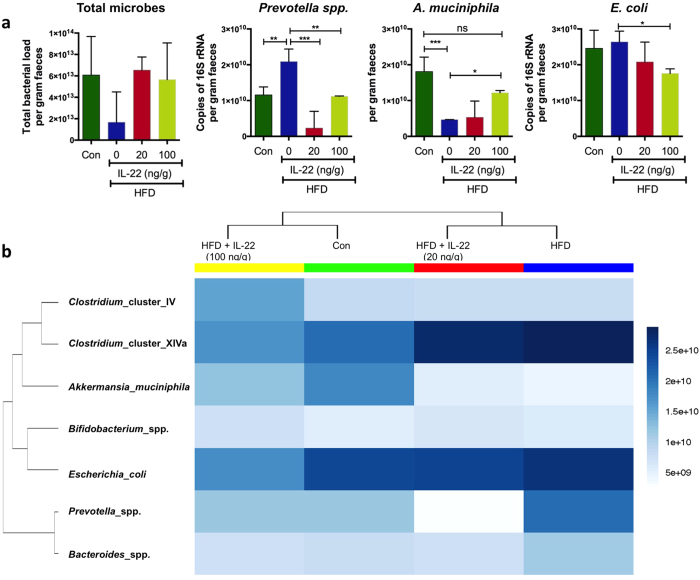
Faecal samples were collected from wild-type C57BL/6 mice fed a HFD or normal chow diet (Con) for 22 weeks and treated with recombinant IL-22 (20 ng/g or 100 ng/g dose) for 4 weeks. (**a**) Levels of total microbial (16S rRNA), *Prevotella* spp, *A. muciniphila* and *E. coli* were determined in the DNA extracted from faecal samples. n = 4 per group. Data presented as mean ± SD. One-way ANOVA with Bonferroni post-test multiple comparisons. *p < 0.05 **p < 0.01 ***p < 0.001. (**b**) Bray-Ward phylocluster analysis was used to determine the overall shifts in microbial population in Con, HFD and HFD mice treated with IL-22. Mean of n = 4 per group.

IL-22 enhances intestinal production of antimicrobial molecules^35^ and IL-22 knockout mice have been shown to have an altered intestinal microbiome capable of transmitting an increased susceptibility to chemically-induced colitis in wild type animals^36^. Therefore, we next examined whether the changes in the microbiome associated with HFD^16^,^37^ were normalised by IL-22-driven improvement in metabolic disease^8^ and intestinal barrier integrity (Fig. 8, Supplementary Fig. 6). Although there were no marked changes in the total microbial load (based on copies of the 16S rRNA gene), consistent with previous studies we observed obesity-associated shifts in the bacterial populations^37^,^38^ and IL-22 treatment normalised these alterations in a dose dependent manner (Fig. 8a,b). More specifically, HFD induced obesity was characterised by an increase in *Prevotella* spp. and a decrease in *Akkermansia muciniphila*, and these changes were largely reversed by treatment of mice with IL-22 (100 ng/g) for 28 days such that no significant differences in the abundance of *Prevotella* spp. and *A. muciniphila* were observed versus mice fed control chow (Fig. 8b). Furthermore, IL-22 treatment resulted in a dose-dependent reduction in the abundance of *Escherichia coli*, which in turn correlated with the decrease in serum endotoxin levels. Collectively, these data suggest that non-esterified fatty acid-induced cellular stress and intestinal inflammation with chronic high fat feeding leads to a diminished mucosal barrier and shifts in microbial populations that can be reversed by IL-22 treatment.

## Discussion

**Figure 9:**
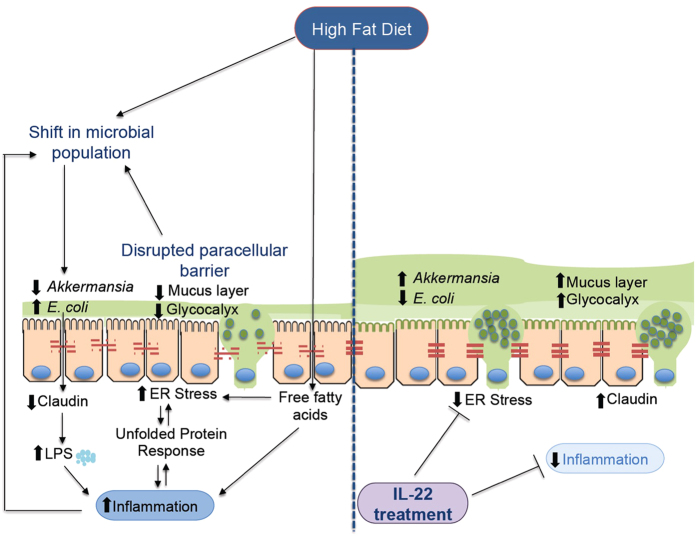
Schematic of the proposed events leading to cellular stress induced by high fat diets and the protective effect of IL-22.

Obesity is linked to the development of many chronic diseases such as diabetes, non-alcoholic steatohepatitis and cardiovascular disease. HFD feeding is known to increase pro-inflammatory cytokines in the intestine and to lead to increased intestinal permeability^10^,^11^,^12^,^39^,^40^. Changes in the gut microbiota and increased leakage of microbial products into the systemic circulation are both considered important elements of the evolution of the metabolic syndrome^13^. Our study shows that high fat diets in mice lead to oxidative and ER stress in intestinal epithelial cells, detrimentally affecting production of the secreted mucosal barrier, and increasing intestinal epithelial paracellular permeability. These stress-induced changes provide a plausible explanation for the altered microbiota, leakage of microbial products and initiation of mucosal inflammation characteristic of human metabolic disease. We also provide a possible mechanistic link by showing that the non-esterified fatty acids which characteristically increase in concentration in obesity are a direct trigger for oxidative and ER stress in intestinal goblet cells. Furthermore, using IL-22 to drive epithelial anti-oxidant defences we demonstrate that intestinal epithelial stress, impaired barrier function and shifts in the microbiome are reversible *in vivo* and therefore potentially amenable to therapeutic intervention (Fig. 9).

Several studies have reported low-grade intestinal inflammation associated with obesity, with a particular focus on the small intestine^41^. Our data shows that a long-term HFD leads to an increase in colonic inflammation particularly in the distal colon. Of note we did not detect any major changes in inflammation or cellular stress in the small intestine, the proximal colon (data not shown) or in the cytokines produced by cultured mesenteric lymph node leukocytes. This could potentially be due to total mucosal mRNA analysis not being sensitive enough to detect the changes in individual immune cell populations demonstrated by others. However, in support of our findings a recent study reported a difference in the shift of the absolute numbers of T_H_1/T_H_17 cells in the colon after 3 weeks of HFD without any changes detected in the small intestine at 3 weeks^13^. In this study, continued high fat feeding lead to changes in the small intestine as well by 12–16 weeks of the diet. Additionally our data corroborates previous studies in which obesity increased the proportion of CD4 + T_H_17 cells systemically and locally within the colon^13^,^40^. It is clear that several different pathways could lead to intestinal inflammation in obesity. It has been proposed that inflammation in the colon precedes the development of metabolic disease following a high fat diet^10^,^41^. Moreover, our study indicates that the low-grade local inflammation and increased serum triglyceride levels within the distal colon is accompanied by cellular stress. We have shown that inflammation can exacerbate protein misfolding and ER stress, using the *Winnie* mouse model of spontaneous colitis^42^. Sustained epithelial cell stress can drive inflammation, by the secretion of chemokines. Therefore, in addition to changes in dietary substrate for the microbiota, the low-grade but persistent inflammation and diminished mucosal barrier could impact the structure-function activity of the microbiota and further contribute to disease. Intestinal bacterial populations are greatly influenced by the host controlled mucosal niche as well as the composition of dietary intake. Supporting our findings, increased levels of *Prevotella* spp. and *E. coli* and decreased *A. muciniphila* have been reported in obese humans and mice^16^,^38^. Importantly, IL-22 treatment of mice was characterised by dose dependent alterations to the structure of the microbiota, despite a continuing HFD, with mice treated with the higher dose of IL-22 most similar to mice fed a control diet. Interestingly, in the absence of endogenous IL-22, the intestinal microbiota has been shown to be colitogenic^36^. The abundance of *A. muciniphila* has been shown to be inversely correlate with hyperglycaemia and increased plasma triglyceride levels in humans^43^,^44^ and in mice feeding *A. muciniphila* improved multiple features of the metabolic syndrome. Consistent with these observations, in our study *A. muciniphila* also accompanied the improvements in metabolic syndrome following IL-22 treatment in mice. It is experimentally difficult to delineate the relative importance and precedence of changes in the host mucosa vs diet-induced changes in microbiota, and it is highly likely that the increase in dietary fat leads to changes in the intestinal barrier via both direct and indirect mechanisms.

With obesity there is an increase in circulating lipids and serum triglycerides and the increase in dietary fat could result in luminal lipids having a direct effect on epithelial cells. Albeit this is more likely to occur in the small intestine, studies suggest that the changes may occur independent of obesity^16^,^21^,^45^ and are therefore suggestive of a more direct impact of NEFAs on the intestinal epithelial cells. In fact, NEFAs can bind to TLR4 and activate signalling pathways^11^,^46^ and here we show that NEFAs can directly initiate ER stress and oxidative stress in intestinal epithelial cells. Activation of the unfolded protein response following a HFD explains the reduction in the transcription of proteins such as *Muc2* and *Tff3*, as well as a decline in goblet cell differentiation factors (*Klf4* and *Spdef*), which is also observed with other ER stressors such as tunicamycin^9^. Although no differences in stored mature Muc2 were observed, we did detect an increase in Muc2 precursor accumulation after 22 weeks of the HFD. Supporting these findings, *in vitro* NEFA-induced UPR also led to a decrease in transcription factors regulating goblet cell differentiation, and to MUC2 misfolding and reduced MUC2 secretion. Decreased MUC2 secretion will result in a thinner mucus layer more easily penetrated by diffusing microbial products, and more easily degraded by mucin-degrading bacteria. This is supported by previous reports that high fat diet-induced obese mice display a ~50% thinner mucus layer in the proximal colon^43^, which could be explained by mucin misfolding and the UPR-activated inhibition of *Muc2* transcription. *In vivo* it is known that pro-inflammatory cytokines such as IL-1β, IFN-γ and TNF-α lead to increasing paracellular permeability. We also noted that NEFA treatment *in vitro* directly decreased transepithelial electrical resistance and *in vivo* we observed decreased claudin-1 staining after 11 weeks of a HFD, which inversely correlated with circulating levels of endotoxins and triglycerides. Therefore, we propose a multifactorial and forward-feeding mechanism mediated by high fat diets and the low-grade inflammation, which leads to MUC2 misfolding, decreased MUC2 secretion as well as a disruption in the glycocalyx and tight junctions leading to a diminished mucosal barrier to microbes which can further exacerbate ER stress and inflammation.

Endogenous mucosal antioxidants, such as glutathione and myeloperoxidase, preserve intestinal homeostasis. Adding to this paradigm, we have discovered that IL-22 is a potent endogenous suppressor of oxidative and ER stress. Leukocytes lack, whereas colonic epithelial cells highly express the heterodimeric IL-22 receptor^47^. We and others have shown that IL-22 therapy in obese mice ameliorates metabolic disease, reducing hyperglycaemia and insulin resistance^8^,^48^. IL-22 has also been shown to upregulate production of antimicrobial peptides in the intestine^48^,^49^. Importantly, as we have previously shown in pancreatic beta cells^8^, in intestinal secretory cells IL-22, even in the absence of stress reduced the expression of *NOS2* and consequently suppresses cellular nitric oxide concentration. Therefore, we hypothesised that IL-22 treatment would resolve high fat diet-induced intestinal cellular stress. As hypothesised, IL-22 treatment down-regulated expression of ER stress and oxidative stress markers and reduced the pro-inflammatory cytokine milieu in the colon. Consistent with the replenishment of the mucus layer (increased Muc2, decreased Muc2 precursor) and epithelial cell barrier integrity (increased claudin-1), we report a marked decrease in serum endotoxin levels. This supports previous *in-vivo* data where gene-delivery of IL-22 has been shown to increase expression of mucins and mucus-producing goblet cells to ameliorate colitis^35^.

Epidemiological and clinical studies reveal that higher-fat diets are associated with a 2.5-fold increased risk of colitis in young adults^50^, moreover 68% of newly diagnosed Crohn’s disease patients were found to be obese in a retrospective study^51^. Using immunocompromised models of spontaneous intestinal inflammation and colitis, multiple investigators have shown an exacerbation of inflammation following a high fat diet. In acute DSS colitis and the *Il10*^−/−^ model of colitis susceptibility, milk-derived fat but not lard fat or unsaturated fat increased the onset, incidence and severity of colitis by promoting proliferation of pathogenic bacterial species exploiting a niche created by specific metabolites of milk fat digestion^52^. In *Muc2*^−/−^ mice a HFD high in palmitic-acid either exacerbated intestinal erosions and mucosal damage in the colon or had a similar effect to regular diet, depending on the esterification of the fatty acid^22^. We demonstrate that HFD feeding exacerbates intestinal inflammation in the *Winnie* mice where ER stress results in spontaneous colitis. *Winnie* mice share similarities with human ulcerative colitis and respond well to drugs used clinically to treat this chronic disease^31^,^53^, therefore providing a strong model of progressive colitis with an intact immune system and without a chemical or infectious trigger being required to initiate disease. The distal colon of the *Winnie* mice on a HFD had widespread colonic damage with mucosal erosions, crypt abscesses and virtually no goblet cells, decreased glycocalyx (and cell surface mucins), disrupted tight junctions and increased markers of oxidative and ER stress. Interestingly, epithelial cell stress was augmented (~70% higher sXBP1) in WT mice kept on the HFD from weaning for 9 weeks compared to WT mice kept on a HFD for 22 weeks from 6–8 weeks of age. These findings may explain the findings from three separate clinical studies, where increased faecal calprotectin and nitric oxide were found in more than 80% of the obese children^54^ but no increases in calprotectin and pro-inflammatory cytokines were detected in lean vs obese adults^55^,^56^. It is apparent that HFDs can exacerbate colitis, however more importantly, our data suggests that early life exposure to HFD has enhanced impact on the intestine and increases the severity of pathology.

Overall, we argue that the cellular stress in intestinal epithelial secretory cells initiated directly by dietary fat will diminish the mucus barrier and begin to activate local immunity. Increasing local inflammatory cytokines will impair epithelial tight junctions which, together with a thinner mucus layer, will increase endotoxin exposure amplifying local inflammation and setting up a forward feeding loop of diminishing mucosal barrier integrity. The altered mucus layer, together with changes in substrate from the diet will dictate unfavourable changes in the microbiota and lead to a cycle of increasing mucosal pathology. Adding to the paradigm of low-grade intestinal inflammation in obesity we show that a HFD accelerated pathogenesis of murine colitis. Important to the prospects of therapy, IL-22 reduces the low-grade intestinal inflammation, restores barrier integrity and leads to a shift in the microbiota back to normal. These findings deepen our understanding of the effect of free fatty acids on the intestinal mucosal barrier integrity, which may help guide nutritional advice for newly diagnosed colitis patients and highlights the beneficial effects of IL-22 therapy beyond the pancreatic islets in obesity.

## Methods

### Animals

Wild type (WT) C57BL/6 (Animal Research Centre, Australia) and *Winnie* mice were housed in a conventional clean, *Helicobacter hepaticus*-free facility on a 12-hour light/dark cycle and fed *ad libitum*. Animals were either fed a HFD, which contained 46% available energy as fat (SF04-027) (Speciality Feeds, Western Australia) or normal chow diet (NCD) (Riverina, Australia), which contained 11% available energy as fat. All experiments were carried out in accordance with approved protocols by the University of Queensland Animal Ethics Committee and were conducted using the Guidelines of the National Health and Medical Research Council. Body weights of mice and diarrhoea and rectal bleeding scores were recorded weekly during the colitis studies. The diarrhoea scoring system can be interpreted as follows: 0 - no diarrhoea: solid stool; 1 - very mild diarrhoea: formed stools that appear moist on the outside; 2 - mild diarrhoea: formed stools that easily submit to pressure applied with forceps; 3 - diarrhoea: no fully formed stools with a mucous-like appearance; 4 -severe, watery diarrhoea: mostly clear or mucous-like liquid stool with very minimal solid present.

### Feeding regimes

3 week HFD: male 6–8 week old WT C57BL/6 mice were fed a HFD or regular control diet for 3 weeks. 9 week HFD: male and female mice 3 week old Winnie mice^7^ on a C57BL/6 background and aged matched WT controls were placed on a HFD or NCD for 9 weeks, 22 week HFD (Additional experimental details and metabolic data was reported previously^8^): Male 6–8 week old WT C57BL/6 mice were fed a HFD or NCD for 22 weeks, and after 18 weeks, high dose (100 ng/g) or low dose (20 ng/g) recombinant IL-22 (Cell Signalling), or phosphate buffered saline (PBS) as a vehicle control, were administered by intra-peritoneal injection twice weekly for 4 weeks. HFD feeding continued during this period - mice were sacrificed after 22 weeks (data shown in Figs 1,2,6 and 7).

### Gene Expression Analysis

The distal colon was snap frozen and homogenised in TRIzol using the FastPrep-24 (MP Biochemicals). RNA was extracted using the High Pure RNA Isolation kit (Roche Industries) according to manufacturer’s instructions, including DNase treatment to remove genomic DNA contamination. RNA concentration was measured using a Nanodrop 1000 Spectrophotometer (Thermo Scientific), followed by complementary DNA synthesis using 1 μg of RNA and the iScript cDNA synthesis kit (BioRad) according to manufacturer’s instructions. The expression of genes of interest (Supplementary Table 1 mouse primer, Supplementary Table 2 human primers) were analysed using quantitative real time PCR (qRT-PCR) using the Via7 Real Time PCR System (Applied Biosystems) using SYBR Premix Ex Taq II (Takara). Ct values were generated and relative quantitation determined by the ΔΔCt method and normalised to housekeeping gene *β-actin* and expressed as a fold difference to the mean of the relevant control samples.

### Antibodies

Mature Muc2 was detected using a polyclonal antibody raised against a murine Muc2 peptide (mMuc2.3)^1^; Muc2 precursor antibody, which detected unglycosylated Muc2^3^, was used to determine Muc2 misfolding (gift from Prof Gunnar Hansson, University of Gothenburg, Sweden). To detect mature human MUC2 a commercially available polyclonal antibody (Santa Cruz Biotechnology, Santa Cruz, CA) was used in combination with the 4F1 monoclonal antibody, which detects unglycosylated MUC2. Claudin-1 mouse monoclonal antibody (Novex #374900) was used to evaluate the density of tight junctions. Polyclonal Grp78 antibody sc-1050 (Santa Cruz Biotechnology) and anti-mouse Ire-1β polyclonal antibody (gift from Prof David Ron, University of Cambridge, Cambridge, England) were used to determine the protein levels of ER stress. Ki-67 antibody (SP6, Thermo Fisher, cat. MA5-14520) was used to identify proliferating cells in the intestine.

### LS174T Cell and Organoid Culture and Treatment

At 70% confluence LS174T cells were treated with 1 mM butyrate, 1 mM propionate, 5 mM palmitate, 15 mM acetate^32^, or a PBS control. After 24 h cells were washed twice with PBS, before RNA was extracted, and after 48 h protein was extracted with 25 mmol/L Tris-HCl, pH 7.4, 0.5% Triton-X-100, 50 mmol/L NaCl with protease inhibitors. Samples were centrifuged at 13,000 g at 4 °C for 20 min and the supernatent was collected. The protein concentration was determined using the BCA Protein Assay Kit (Pierce #23225) as per the manufacturer’s instructions. 700 μg of protein was subsequently reduced for 15 min using 50 mmol/L dithiothreitol at 95 °C and carboxymethylated for 30 min using 0.125 mol/L iodoacetamide at room temperature in the dark, before electrophoresis on a 1% (w/v) agarose gel at 15 V for 16 h, then vacuum transferred onto a nitrocellulose membrane and briefly PBS washed before blocking in Odyssey® blocking buffer (PBS) (LiCor) for 2 h at room temperature. The membrane was then probed with Muc2 (1:500 Santa Cruz sc-15334) and Muc2 precursor (1:1000) antibodies in Odyssey® blocking buffer overnight at 4 °C. The membrane was washed three times with TBS plus 0.05% Tween20 buffer then incubated with Dylight® 680 and 700 conjugated secondary antibodies (1:20,000 Cell Signaling Technology), in TBS plus 0.05% Tween20 buffer for 1 h at room temperature. The membrane was washed a further three times with TBS plus 0.05% Tween20 buffer, and fluorescence was measured using Odyssey® Infrared Imaging System (LiCor model #9120) with Image Studio^Tm^ Software (LiCor v.3.0.30). For human organoid cultures, ~5 cm of colonic biopsy was washed with ice cold PBS and incubated with 8 mM EDTA for 1 h on ice. Supernatant was discarded and chunks extracted were allowed to settle before several washes with PBS. Crypts were counted, resuspended in PBS/0.1% BSA, plated in Matrigel with 75% condition media from L-WRN cells (a kind gift from Thaddeus Stappenbeck) and 25% Advanced DMEM containing 10% FBS and Penstrep, 10 nM Rock inhibitor, 10 nM SB431542 and 5 nM CHIR99021. Crypts were allowed to grow and recover for 2 days before treatment with 5 mM palmitate in the precence or absence of 50 μg/mL of IL-22 for 24 h. 0.1% BSA was used as a control.

### Histology, Immunofluorescence Microscopy, and Assessment of Histologic Colitis

The whole colon, cecum, and distal 5 cm of the small intestine were rolled, fixed in 10% neutral buffered formalin, and paraffin embedded and sectioned. Immunohistochemical and immunofluorescent staining methods previously described were used to determine the levels of Muc2, Muc2 precursor and claudin-1^9^,^57^. Apoptotic cells in tissue sections were detected using TUNEL staining (Roche), used in accordance with instructions provided by the manufacturer. Blind assessment of histologic inflammation (aberrant crypt architecture, increased crypt length, increased leukocyte infiltration, neutrophil counts, goblet cell depletion, crypt abscesses, and epithelial cell damage and ulceration) for small intestine, cecum, proximal colon, mid-colon, and distal colon was performed by S.Z.H., M.A.M. and M.G as previously described (maximum combined score of 25). Diaminobenzidine stained slides were dehydrated in an ethanol gradient and incubated in xylene before cover slipped (Pertex® Mounting Medium), and scanned (Olympus VS120) at 40x magnification. Fluorescent slides were cover slipped manually using Prolong® Gold (Life Technology) then imaged (Olympus Epi upright microscope) at 20x and 60x magnification. Representative images illustrating colitis score or mucin quantification were taken from the median scored sample from each group.

### Histological Mucin Quantification

For each blinded sample, two representative images of the mid colon were analysed using ImageJ v1.46 (W. Rasband, Open Source). The mid colon was chosen because it is reflects both proximal and distal colon tissue analysed by qRT-PCR and contains both lineages of intestinal goblet cells. For each image, the total pixel area of a selection of at least 10 colonic crypts was measured. Intracellular Muc2 or glycoprotein was then isolated by colour contrast manipulation and the pixel area measured. Percentage volume of Muc2/glycoprotein as a proportion of total crypt volume was then calculated for both images and the weighted average based on the number of crypts sampled per image was determined. For claudin-1 staining, the intensity of area stained was measured using ImageJ v1.46 in 4 fields of view per animal.

### Cytokine Protein Analysis

Mesenteric lymph nodes were were isolated and cells were resuspended at 5 × 10^6^ cells/mL in RPMI 1640 with 10% fetal bovine serum, 2 mmol/L L-glutamine, 100 U/mL penicillin, and 100 μg/mL streptomycin. Anti-CD3 and anti-CD28 were used for stimulate cytokine secretion for 24 hours at 37 °C and 5% CO_2_. Cell-free supernatants were stored at −80 °C; enzyme-linked immunosorbent assay for TNF-α, IL-1β, and IL-17a according to the manufacturer’s instructions (R&D Systems) to determine the concentration of these cytokines.

### Epithelial cell isolation and Western Blot analysis

Distal colon was isolated from mice kept on a normal chow diet or a high fat diet in HBSS without Ca^2+^ and Mg^2+^ (Invitrogen). The intestine was cut longitudinally and cut in 5 mm lengths. To remove the mucus the tissue was incubated with 10 mM Dithiothrethiol in HBSS for 15 mins. The supernatant was removed and 0.8 mM EDTA was added, and incubated for 30 mins with gentle shaking and rotation. The supernatant was removed and HBSS was added. The tissue was shaken vigorously to isolate the epithelial crypts and the supernatant containing crypts was centrifuged at 40×g for 5 minutes to remove single cells. The crypts were then treated for 20 min on ice with RIPA cell lysate buffer (RIPA buffer: 50 mM Tris-HCI, pH 7.5, 150 mM NaCI, 1.0% Nonidet P-40, 0.1% sodium deoxycholate supplemented with complete protease inhibitor cocktail and phosphate stop) and mixed using a vortex every 5 min. The resulting lysates were centrifuged at 16,000 g for 20 min at 4 °C to remove cell debris and stored at −80 °C. BCA assay was conducted to determine the protein concentration, protein was analysed by SDS-PAGE analysis.

### Microbial Assessment

All mice were littermates and were co-housed together for 2 weeks prior to being places on the diets. All groups of mice were randomly allocated to treatments within cage groups to eliminate cage effects. DNA was extracted from faeces collected from 22 week experiment as described above using the Maxwell 16 Instrument as described previously^58^. Standard curves were generated from all microbial genes (described in Supplementary Table 3) and copy numbers were determined using Endmemo (http://endmemo.com). Heatmap of qPCR data was generated using the heatmap function of the NMF package implemented in R^59^. Ward’s minimum variance method was used for hierarchical clustering of the computed distance matrix for samples based on qPCR values using Bray Curtis dissimilarity measures in the vegan package implemented in R^60^.

### Endotoxin and Triglyceride Assay

Serum collected from mice at the end of the experiment was analysed using the QCL-1000™ Endpoint Chromogenic LAL Assay (Lonza) to determine endotoxin levels and Serum Triglyceride Quantification Colorimetric Kit (Cell Biolabs, STA-396) to determine the triglyceride levels.

### Statistical Analyses

Statistical analysis was performed using GraphPad Prism version 5.01 (GraphPad Software, Inc, La Jolla, CA). Differences between groups were assessed by analysis of variance with Bonferroni post hoc tests after confirmation of normal distribution by probability plots. Box plots show median, quartiles, and range unless stated otherwise.

## Additional Information

**How to cite this article**: Gulhane, M. *et al*. High Fat Diets Induce Colonic Epithelial Cell Stress and Inflammation that is Reversed by IL-22. *Sci. Rep.* **6**, 28990; doi: 10.1038/srep28990 (2016).

## Supplementary Material

Supplementary Information
