# Supplementary material for: High Fat Diets Induce Colonic Epithelial Cell Stress and Inflammation that is Reversed by IL-22
Source: Sci Rep. 2016 Jun 28;6:28990. doi: 10.1038/srep28990 (PMC4924095; doi:10.1038/srep28990)
Supplement: Supplementary Information [file srep28990-s1.pdf]

Supplementary Information

High Fat Diets Induce Colonic Epithelial Cell Stress and Inflammation that is Reversed by IL-22

Max Gulhane[1], Lydia Murray[1], Rohan Lourie[1], Hui Tong[1], Yong H. Sheng[1], Ran Wang[1], Alicia Kang[2], Veronika Schreiber[1], Kuan Yau Wong[1], Graham Magor[3], Stuart Denman[4], Jakob Begun[1], Timothy H. Florin[1], Andrew Perkins[3], Páraic Ó Cuív[2], Michael McGuckin[1] and Sumaira Z. Hasnain[1]

(1) Immunity, Infection and Inflammation Program, (2) University of Queensland Diamantina Institute, Translational Research Institute (3) Blood and Bone Diseases Program, Mater Research Institute - The University of Queensland, Translational Research Institute, Brisbane, Australia, (4) The Commonwealth Scientific and Industrial Research Organization, St Lucia, Brisbane, Australia

The authors declare no conflict of interest

Correspondence

Dr. Sumaira Z. Hasnain

Mater Research Institute – University of Queensland, Translational Research Institute, 37 Kent St, Woolloongabba, Qld 4102

t: +61-7-34436939, f: +61-7-31632550 ; E: sumaira.hasnain@mater.uq.edu.au

ABBREVIATIONS

IBD, inflammatory bowel disease; KLF4: Kruppel-like factor 4 ER, endoplasmic reticulum; UPR, unfolded protein response; sXBP1, spliced X-box binding protein 1; PERK, protein kinase RNA-like ER kinase; ATF6, activating transcription factor 6; IRE-1, inositol requiring enzyme-1; NEFA, Non-esterified fatty acid; ROS, reactive oxygen species; iNOS, inducible nitric oxide synthase.

Supplementary Figure Legends:

Supplementary Figure 1: Wild-type C57BL/6 mice were fed a high fat diet (HFD) or regular control diet (Con) for 3 weeks (n=6-7 per group), 11 weeks (n=5-6 per group) or 22 weeks (n=8-12 per group). **(a)** Final body weights (in grams) of mice at the time of sampling. **(b)** TNF-α, IL-1β, and IL-17a secreted by anti-CD3/anti-CD45–stimulated leukocytes isolated from mesenteric lymph nodes of control and mice kept on a HFD for 22 weeks. The red dashed line depicts the limit of detection. mRNA level of proinflammatory cytokines **(c)** *Il4*, **(d)** *Il13* and **(e)** *Il23* was determined by qRT-PCR in the distal colon. Normalized to *B-actin* and expressed as a fold change of the respective controls. n = 6-8. Data presented as mean ± SEM, One way ANOVA with Bonferroni post-test. *p<0.05 **p<0.01 ***p<0.001.

Supplementary Figure 2: Wild-type C57BL/6 mice were fed a high fat diet (HFD) or regular control diet (Con) for 3 weeks (n=6-7 per group), 11 weeks (n=5-6 per group) or 22 weeks (n=8-12 per group). **(a, c, e)** Volumetric analysis of mature mucin as a percentage of crypt area as depicted by Periodic Acid Schiff's-Alcian blue staining shown in Fig 2a. **(b, d, f)** ImageJ analysis of area stained with Muc2 antibody using immunohistochemistry. qRT-PCR was used to determine the levels of **(g)** goblet cell protein trefoil factor 3 (*Tff3*) and **(h)** goblet cell differentiation factor *Spdef*. Data is normalized to *B-actin* and expressed as a fold change of the respective controls. **(i)** Immunohistochemistry and area stained analysis with Muc2 precursor antibody was used to assess the Muc2 misfolding. **(j)** Immunofluorescence was used to determine the levels of claudin-1 (original images depicted in Fig. 2e) **(k)** Crypt length measurements (μM). Data presented as mean ± SEM or box plots with whiskers show median. Q1, Q3 and min/max, One-Way ANOVA with Bonferroni post-test. *p<0.05 **p<0.01 ***p<0.001.

Supplementary Figure 3: Colon weight/length ratio from wild-type C57BL/6 or *Winnie* mice fed a high fat diet (HFD) or normal chow diet (NCD) for 9 weeks following weaning (3 weeks of age). One-Way ANOVA with Bonferroni post-test. ***p<0.001 compared to NCD. n = 5-8.

Supplementary Figure 4: **(a)** Serum triglyceride levels in control mice compared to mice kept on HFD for 3, 1 and 22 weeks. n = 6-8. LS174T cells were treated with Control BSA, 0.5mM Palmitate or 1 mM butyrate, 1 mM proprionate, 15 mM acetate or 50 ng/mL of IL-22 for 24 hours. mRNA levels of ER stress markers **(b)** *GRP78*, **(c)** *spliced XBP1,* goblet cell differentiation factor **(d)** *KLF4*, major component of goblet cells **(e)** *MUCIN-2* and the component of the glycocalyx cell surface **(f)** *MUCIN-1*, was determined by qRT-PCR. qRT-PCR was used to determine the expression of oxidative stress marker *Nos2* **(g)**, and Griess Assay was used to determine the changes in oxidative stress protein Nitrite **(h)**. qRT-PCR data is normalized to mean expression of *β-Actin* and expressed as a fold change compared to BSA controls. Statistics: n= 8 per group (2 individual experiments). One way ANOVA with Bonferroni post-test; *p<0.05 **p<0.01 ***p<0.001.

Supplementary Figure 5: Wild-type C57BL/6 mice were fed a high fat diet (HFD) or normal chow diet (Con) for 22 weeks. After 18 weeks, recombinant IL-22 was administered at 20 ng/g or 100 ng/g i.p for 4 weeks. **(a)** Colon weight/length ratio was determined as a measure of inflammation. **(b)** Staining with mature Muc2 antibody, imageJ analysis depicting the intracellular Muc2 staining as a percentage of crypt area. **(c)** Crypt length measurements (μM). **(d)** Staining with Ki67 antibody to assess a change in proliferation. **(e)** Representative images of epithelial cell apoptosis detected by triphosphate nick-end labeling (TUNEL) staining in the colon. Negative control shows background autoflourescence and positive control shows apoptosis in epithelial cells digested with DNAse. **(f)** Staining with Grp78 antibody, showing an increase in ER stress in HFD mice and a reduction with IL-22 treatment. One-Way ANOVA with Bonferroni post-test. ***p<0.001 compared to NCD. n = 8-12.

Supplementary Figure 6: Levels of *Bifidobacterium* spp*., Clostridium* cluster XIVa*, Bacteroides* spp*. and Clostridium* cluster IV were determined in the DNA extracted from faecal samples. n = 4 per group. One way ANOVA with Bonferroni post-test; *p<0.05 **p<0.01 ***p<0.001.

Supplementary Figure 1

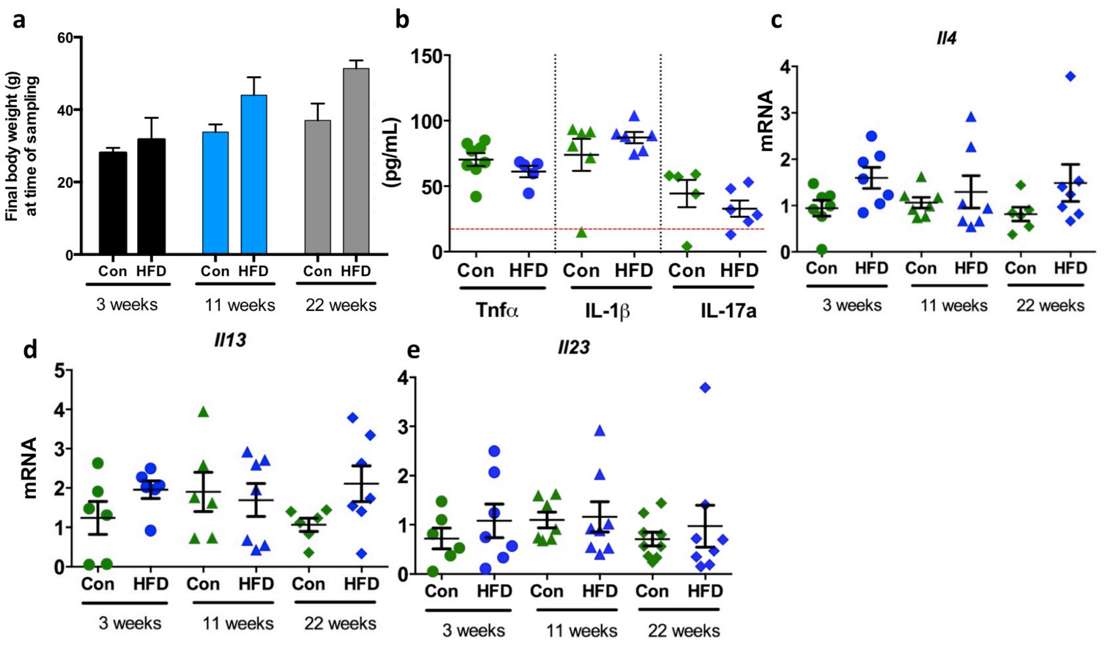

Supplementary Figure 2

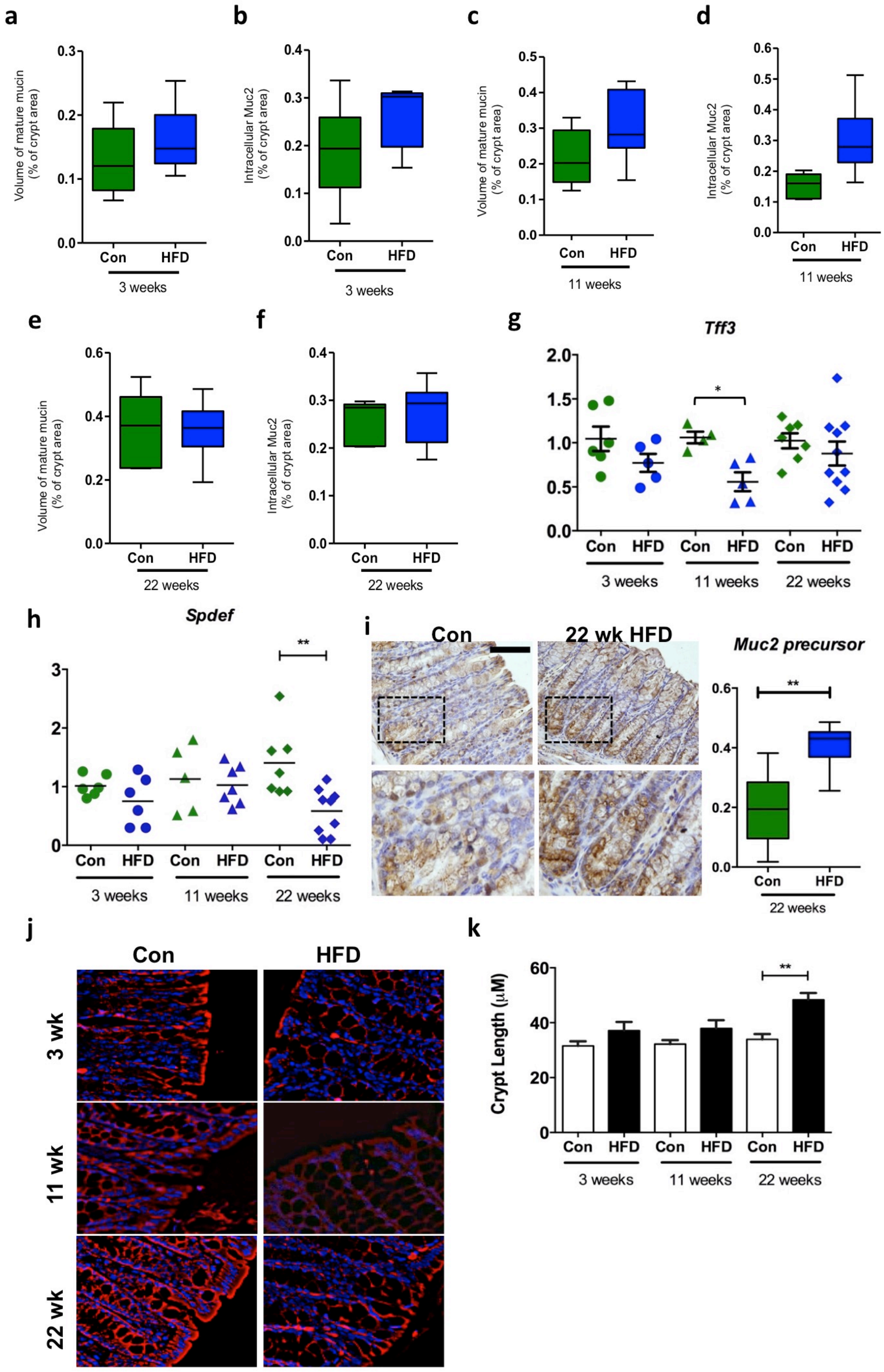

Supplementary Figure 3

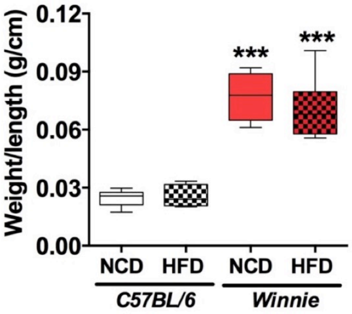

Supplementary Figure 4

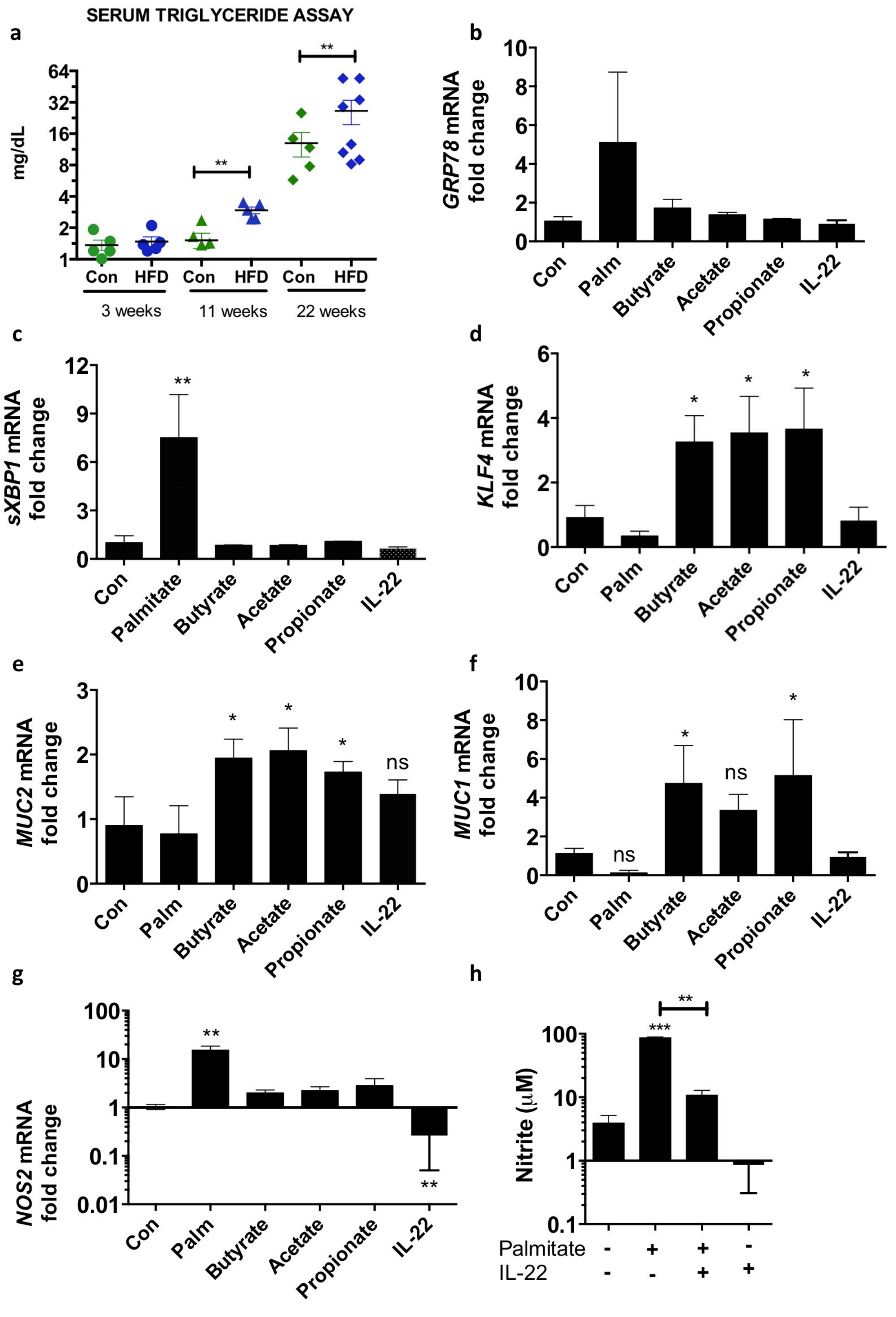

Supplementary Figure 5

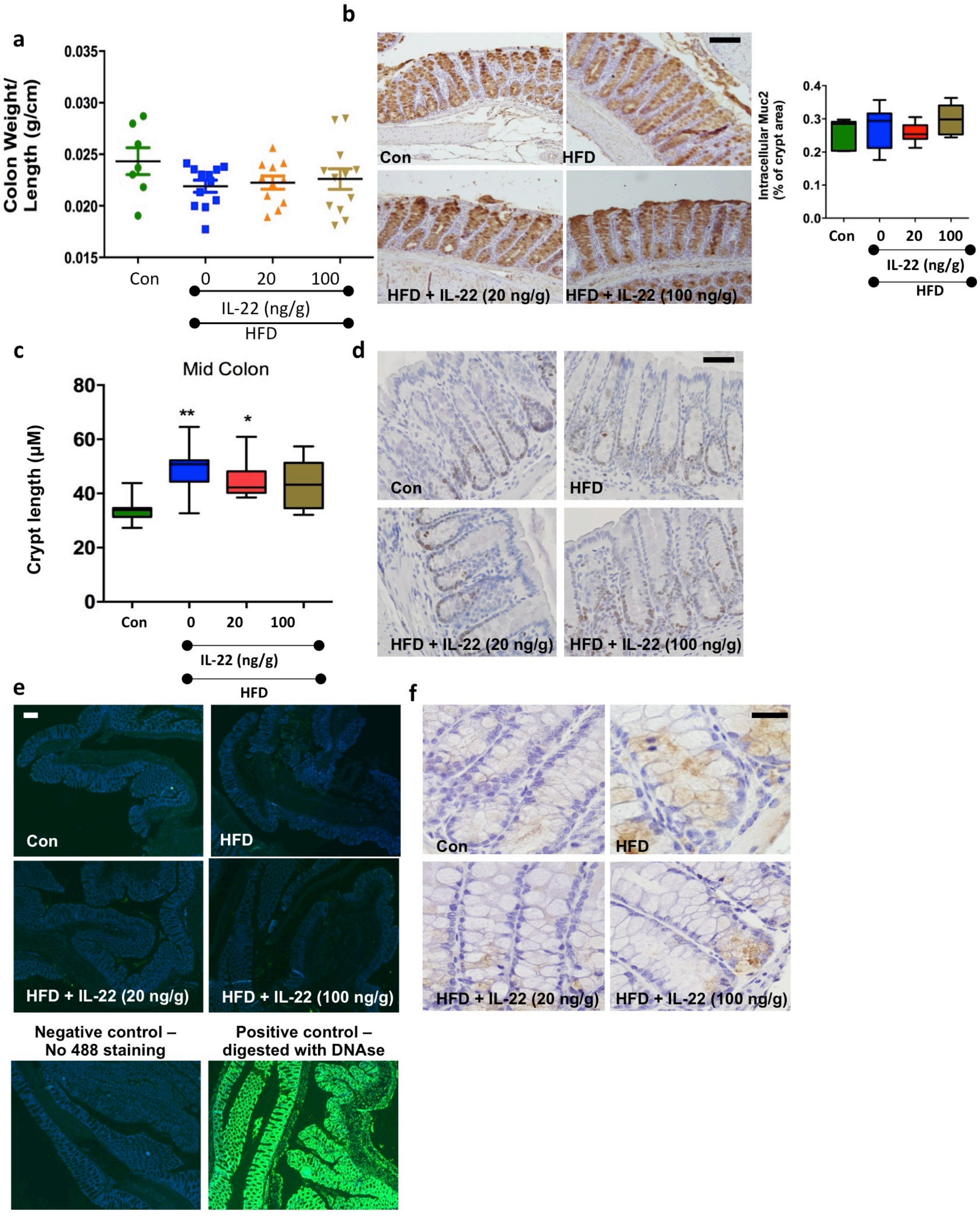

Supplementary Figure 6

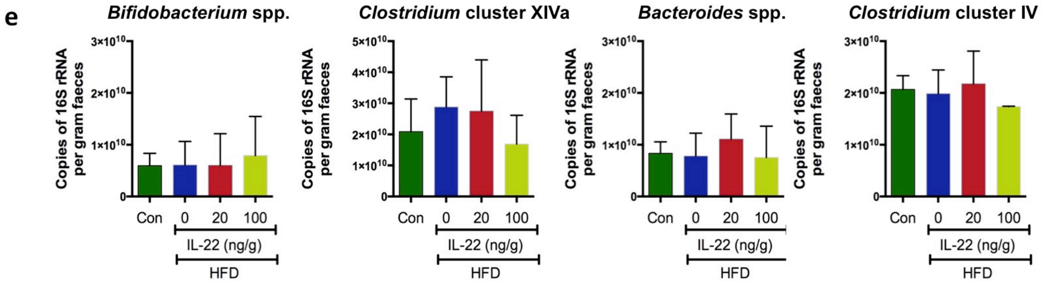

Supplementary Table 1 – Mouse Primer sequences

**Target gene**	**Forward Primer (5'–3')**	**Reverse Primer (5'–3')**
β-actin	AGCACTGTGTTGGCATAGAGGTC	CTTCTTGGGTATGGAATCCTGTG
Edem1	AAGCCTGCAATGAAGGAGAA	CTATCAGCACCTGCAGTCCA
Grp78	TGCTGCTAGGCCTGCTCCGA	CGACCACCGTGCCCACATCC
Il1β	CAACCAACAAGTGATATTCTCCATG	GATCCACACTCTCCAGCTGCA
Il17a	CTCCAGAAGGCCCTCAGACTAC	AGCTTTCCCTCCGCATTGACACAG
Il4	GAGCTCGTATGTAGGGCTTC	GCCCGAAAGAGTCTCTGC
Il22	CCGAGGAGTCAGTGCTAAGG	CATGTAGGGCTGGAACCTGT
Il23	TGTCACGGAGGAATCACAAG	TGTGCATGTGAAGAGTTTGGA
Klf4	AGCCACCCACACTTGTGACTATG	CAGTGGTAAGGTTTCTCGCCTGTG
Muc2	CCATTGAGTTTGGGAACATGC	TTCGGCTCGGTGTTCAGAG
Muc1	CCCCCCTGGCACATACTGGG	ACCTCACACACGGAGCGCCAG
Muc4	GCTCAAGTTGACAAGGAGCAGAGC	GGAGGACAAAAGAAGGCGTGGCC
Muc13	GCCAGTCCTCCCACCACGGTA	CTGGGACCTGTGCTTCCACCG
Nos2	CAGCTGGGCTGTACAAACCTT	CATTGGAAGTGAAGCGTTTCG
Spdef	GGTGCCTGCTACTGTTCCCAGATG	AAAGCCACTTCTGCACGTTACCAG
sXbp1	GAGTCCGCAGCAGGTGC	CAAAAGGATATCAGACTCAGAATCTGAA
Tff3	CCTGGTTGCTGGGTCCTCTGG	GTCTCCTGCAGAGGTTTGAAGC
Tnfa	CATCTTCTCAAAATTCGAGTGACAA	TGGGAGTAGACAAGGTACAACCC

Supplementary Table 2 – Human Primer sequences

Target gene	Forward Primer (5'–3')	Reverse Primer (5'–3')
B-ACTIN	CCTGTACGCCAACACAGTGC	ATACTCCTGCTTGCTGATCC
GRP78	GCCTGTATTTCTAGACCTGCC	TTCATCTTGCCAGCCAGTTG
KLF4	AGAGGAGCCCAAGCCAAAGA	CAGTCACAGTGGTAAGGTTTCTC
MUC1	CCCCTATGAGAAGGTTTCTGC	ACCTGAGTGGAGTGGAATGG
MUC2	CAGCACCGATTGCTGAGTTG	GCTGGTCATCTCAATGGCAG
NOS2	CACTCAGCTGTGCATCGAC	CAGTTCCCGAAACCACTCGT
sXBP1	GAGTCCGCAGCAGGTGC	CAAAAGGATATCAGACTCAGAATCTGAA

Supplementary Table 3 – Bacterial Primer sequences

Target gene	Forward Primer (5'–3')	Reverse Primer (5'–3')
Universal 16S rRNA	CGGCAACGAGCGCAACCC	CCATTGTAGCACGTGTGTAGCC
Akkermansia muciniphila	CAGCACGTGAAGGTGGGGAC	CCTTGCGGTTGGCTTCAGAT
Clostridium cluster IV	TTACTGGGTGTAAAGGG	TAGAGTGCTCTTGCGTA
Clostridium cluster XIVa	AAATGACGGTACCTGACTAA	CTTTGAGTTTCATTCTTGCGAA
Prevotella spp.	CACRGTAAACGATGGATGCC	GGTCGGGTTGCAGACC
Bacteroides spp.	AAGGTCCCCCACATTGG	GAGCCGCAAACTTTCACAA
Bifidobacterium spp.	GGGTGGTAATGCCGGATG	CCACCGTTACACCGGGAA
E. coli	GGAAGAAGCTTGCTTCTTTGCTGAC	AGCCCGGGGATTTCACATCTGACTTA
